# The impact of methylprednisolone and rituximab on podocyte injury caused by puromycin aminonucleoside

**DOI:** 10.3389/fcell.2025.1504834

**Published:** 2025-12-10

**Authors:** Li Wang, Manman Zhao, Jialiang Zhu, Ran Hua, Ying Zhu, Fang Deng

**Affiliations:** 1 Department of Pediatrics, The First Affiliated Hospital of Anhui Medical University, Hefei, Anhui, China; 2 Department of Nephrology, Children’s Hospital of Anhui Medical University (Anhui Provincial Children’s Hospital), Hefei, Anhui, China

**Keywords:** rituximab, methylprednisolone, TRPC6, calcium influx, podocyte

## Abstract

**Introduction:**

To explore how MP and RTX impact TRPC6's expression and localization, and assess MP's and RTX's effects on podocyte injury and recovery.

**Methods:**

MPC5 cells were simultaneously grown alongside a control group and under various conditions: exposure to puromycin aminonucleoside (PAN) stimulation, treatment with methylprednisolone (MP), and treatment with rituximab (RTX), and a combined treatment with both MP and RTX.

**Results:**

At 8, 24, and 48 h, CCK-8 assay showed that PAN (50 μg/mL) had a decrease in cell viability and an increase in cell death, and it could be used as the optimum concentration to induce podocyte injury; MP (100 ng/mL) and RTX (100 μg/mL) maintained cell viability and had minimal impact on cell morphology, and they were the best concentrations. Following 24 and 48-h exposure to MP or RTX, there was a decrease of 30%–50% in apoptosis rates by flow cytometry in comparison to the group stimulated with PAN, accompanied by a substantial reduction in nearly 10%–60% of TRPC6 mRNA and 5%–20% of protein levels which were measured using qRT-PCR and western blot analyses, akin to the observed decrease in levels of IL-1β and IL-18. Additionally, calcium entry showed considerable reductions after 8, 24, and 48 h of MP treatment relative to the PAN-stimulation group, paralleling the effect seen with 24-h RTX treatment.

**Discussion:**

Therefore, MP and RTX safeguarded podocytes, and averted proteinuria by decreasing podocyte apoptosis, diminishing TRPC6 mRNA and protein levels, and suppressing inflammatory markers and calcium entry.

## Introduction

Nephrotic syndrome (NS) is a common clinical glomerular disease, and massive proteinuria is its main clinical indication. Central to NS is immune system dysfunction, particularly the dysregulation of podocytes and inflammation factors that damage the glomerular filtration barrier (GFB), mainly composed of three layers, namely, glomerular endothelial cells, basement membrane, and podocytes, respectively, from inside to outside (Vivarelli et al., 2023). Release of proteinuria or NS are symptoms of podocytopathies, glomerular disease caused by direct or indirect podocyte injury, which link to immune system (Myette et al., 2025). Nephrin, the major podocyte antigen, contributes to renal injury through the production of autoantibodies, and prevents macromolecular proteins carrying the same charge properties from leaking out of the filtration barrier (Al-Aubodah et al., 2025; Duan et al., 2025; Qu and Jiao, 2023; Colucci et al., 2022; Meng et al., 2025).

As the last barrier to proteinuria, podocytes are the key target cells in the whole process of occurrence and development of NS (Vivarelli et al., 2023; Meliambro et al., 2024). Structural podocyte abnormalities, such as abnormalities in actin cytoskeleton or slit diagram (SD), can cause foot processes (FPs) fusion, and affect the integrity of the GFB, indicating that podocyte injury and proteinuria, which account for up to 90% of cases of worsening kidney function worldwide (Meliambro et al., 2024; Loreth et al., 2025). Nevertheless, podocyte foot process morphology has diagnostic value in differentiating diabetic nephropathy (DN) and minimal change disease (MCD) (Li et al., 2025).

Moreover, podocytes are postmitotic cells and have a very limited capacity for self-renewal (Loreth et al., 2025; Haydak and Azeloglu, 2024). Podocyte loss, whether due to detachment or cell death, results in irreversible damage and scarring of the renal filtration units (Meliambro et al., 2024; Haydak and Azeloglu, 2024). Podocyte injury has been reported to be associated with intracellular calcium (Ca^2+^) overload (Ilatovskaya et al., 2025). Transient receptor potential ion channel 6 (TRPC6) has been recognized as a novel SD protein involved in maintaining the structural stability of the podocyte skeleton and regulating Ca^2+^ homeostasis (Lu et al., 2019). It has been confirmed by TRPC6-specific inhibitor through attenuating the degradation of podocyte structural proteins, inhibiting fluorescence intensity of intracellular Ca^2+^, and podocyte apoptosis, resulted in podocyte injury and recovery *in vitro* (Feng et al., 2022). It was also shown that TRPC6 gene variation in glomerular human glomerular diseases, including MCD, FSGS, and immune complex associated glomerulonephritis (Sun et al., 2021), and TRPC6 overexpression in podocytes correlate with decreased calpastatin expression, autophagy blockade, and podocyte injury in DN (Salemkour et al., 2023). Therefore, TRPC6-directed therapy is therefore currently being targeted for treatment for podocytopathies.

Corticosteroids are the cornerstone of the treatment of NS. However, 5%–15% children who do not respond to a cycle of oral steroids, and 55%–60% have frequent relapses and require repeated or ongoing use of glucocorticoids, therefore, most of them require steroid-sparing immunosuppressive agents, including calcineurin inhibitors, rituximab (RTX) (Vivarelli et al., 2023). Aside from depleting CD20 B cells, RTX binds to podocyte SMPDL3b and has non-immunological effect on podocytes by reducing podocyte injury and apoptosis, increasing cell adhesion, and stabilizing actin cytoskeleton, contributing to its effectiveness in reducing proteinuria (Jeruschke et al., 2022; Aslam and Koirala, 2023). Furthermore, the depletion of antigen-presenting B cells by RTX may target B-cell survival signaling through the BAFF/APRIL pathway, restore the balance between autoreactive T cells and regulatory T cells, and suppress interleukin (IL)-13 secretion by Th2 cells in autoimmune diseases (Lin et al., 2024).

Clinical studies have demonstrated that combining RTX treatment with methylprednisolone (MP) pulse therapy might be more effective in reducing proteinuria and relapse rates in patients suffering from NS than RTX alone, however, the precise pharmacological mechanism of RTX and MP are not well understood yet (Chan and Tullus, 2021; Chan et al., 2023; Yokota et al., 2024; Nozu et al., 2024). *In vivo* and vitro, TRPC6-targeted dexamethasone (Dex) nanobubles could alleviate podocyte apoptosis and inflammation, suggesting that TRPC6 might be an ideal guiding target for glucocorticoids-based renal therapy (Wu et al., 2025). Puromycin aminonucleoside (PAN) is used mainly *in vitro* and vivo, not in clinic, and there is no standard for the safe doses at human level for its potential toxicity. Furthermore, PAN treatment could significantly disrupt the cytoskeletal architecture of cultured mouse podocytes, and reduce the formation of focal adhesions and stress fibers. Interdigiting intercellular junctions were replaced by dot-like structures with accumulated filamentous actin (Huang et al., 2025). In this study, we utilized a podocyte injury model with PAN treatment to explore how MP and RTX impact TRPC6’s expression and localization, and assess MP’s and RTX’s effects on podocyte injury and recovery.

## Materials and methods

### Cell culture

Cell line mouse podocyte clone 5 (MPC5) was procured from the Chinese Academy of Sciences Cell Bank (Shanghai). The podocytes underwent cultivation in 1% penicillin-streptomycin (Beyotime, Biological Industries, China, Israel) with an added 10% fetal bovine serum (FBS) (Viva Cell, Shanghai, China), and were carried out at a steady temperature of 37 °C inside an incubator enriched with 5% carbon dioxide. The trials were segmented into five groups: the control group, and PAN (Bioss, China) stimulation group. Both RTX (Roche, Switzerland) and PAN incorporated to engage with the podocytes. Both MP (Manufacturing Belgium NV, Pfizer) and PAN were solubilized for podocytes engagement. Correspondingly, PAN along with RTX and MP were introduced and homogenized to facilitate interaction with the podocytes.

### CCK-8 assay

Various amounts of podocytes per well were dispensed into 96-well plates, each containing a 100 µL sample, and allowed to settle for 12 h at 37 °C. Firstly, 10 µL of the CCK-8 solution (BA00208, Beyotime, China) was introduced to each well and the plates were incubated at 37 °C for 1 h. The absorbance at 450 nm of the plates was then measured every hour for a total of six times using a spectrophotometer. After determining the optimal cell density, podocytes were plated again in 96-well dishes at this concentration, using 100 µL volume for each well for a duration of 12 h. Secondly, varying levels of PAN, RTX, and MP were applied for a duration of 1 h, succeeded by the addition of a 10 µL CCK-8 mixture, which was then cultivated at 37 °C for an additional 2 h before the optical density was measured. Once an appropriate dosage was established, administration occurred over various time spans (8, 24, or 48 h). Subsequently, podocytes received PAN at the determined optimal concentration and duration, with MP and RTX being administered either in conjunction or not.

### Quantitative real-time polymerase chain reaction (qRT-PCR)

Following the guidelines provided by the RNAgents Total RNA Isolation System’s manual (AG11701, ACCURATE BIOLOGY, China), cellular RNA was isolated. The RNA was harvested from the cells employing the prescribed protocol of the RNA extraction kit (AG21102). Subsequently, reverse transcription was carried out using the ACCURATE BIOLOGY®RT Reagent Kit (AG11706) with cDNA eraser. Primers tailored for GAPDH (forward 5’--3′ and reverse 5’--3′) (Table 1) were utilized to conduct a one-step real-time PCR assay.

**TABLE 1:** Primers tailored for GAPDH (5′–3′).

Gene expression	Forward 5’--3′	Reverse 5’--3′
TRPC6	GGAAGCCATTGGCAGAACCT	CAGGGGCAGCCTTTAGAGAG
β-actin	TGTGTCCGTCGTGGATCTGA	TTGCTGTTGAAGTCGCAGGAG

### Flow cytometry

The cellular collection was accomplished through trypsinization, omitting EDTA, followed by a duo of phosphate-buffered saline (PBS) (PB180327, PH = 7.4) rinses. Subsequently, half a milliliter of the binding buffer solution was dispensed into each well of a 96-well plate. The cells underwent incubation in obscurity with a mixture of 5 μL each of Annexin V-EGFP (BB-4102) and propidium iodide for a duration ranging from 10 to 20 min at 25 °C. Post-incubation, a BD FACSVerse flow cytometer (BD Bioscience, located in San Jose, CA, United States) was employed for the analytical process. Apoptosis was quantified using FCM and annexin V-FITC/PI.

### Western blot analysis

MPC5 was prepared with a radioimmunoprecipitation (RIPA) lysis containg phenylmethanesulfonyl fluoride (PMSF). After blocking, the polyvinylidene fluoride (PVDF) membranes were washed with Tris-buffered saline containing 0.1% Tween-20 (TBST) thrice and incubated overnight at 4 °C with primary antibodies (TRPC6, ab105845, 1:1 000, Abcam; β-actin, ab8227, 1:5 000). Next, the PVDF membranes were washed with TBST thrice and incubated with horseradish peroxidase (HRP, 1:5 000, Beyotime, China)-conjugated secondary antibodies at room temperature for 1 h. Then bands were detected by Tanon 5200 image analysis system (Tanon, Shanghai, China). Quantitative densitometry was performed using ImageJ. Intensity values expressed as the relative protein expression were normalized to β-actin.

### Assessment of TRPC6 localization in podocytes using immunofluorescence labelling

The cover glass was nearly fully coated with the cells, which were then stabilized using ice-cold acetone and further preserved with 4% paraformaldehyde for a duration of 15 min at 4 °C away from light. Subsequently, the cells underwent two rounds of PBS rinsing, followed by a period of incubation with both primary and secondary antibodies. Following a sequence of five additional PBS washes, the cellular nuclei were stained with -diamidino-2-phenylindole (DAPI) (C1006) sourced from Beyotime in Shanghai, China. Photographs were captured through the oil immersion lens of a Zeiss LSM 880 laser scanning confocal microscope (Leica, Germany), utilizing the FITC (green) filter at a 488 nm excitation wavelength, and subsequently examined via computational analysis.

### Enzyme-linked immunosorbent assay (ELISA)

Levels of IL-1β (E-EL-M0037c, Elabscience, China) and IL-18 (E-EL-M0730c, Elabscience, China) in the supernatants of cultured cells were measured with industry-standard ELISA kits supplied by Elabscience Biotechnology Co. based in Wuhan, China. The protocols were carried out in strict adherence to the guidelines provided by the kit’s producer.

### Calcium imaging

Glass coverslips measuring 22 mm across, with podocytes adhered to them, were subjected to a 30-min incubation period at 25 °C away from light, in the presence of 5 μΜ Fura-2AM (S1052). Excess Fura-2AM was washed out by pumping normal physiological saling solution (NPSS) containing 4.09 g NaCl, 0.1862 g KCl, 0.0555 g CaCl_2_, 0.0475 g MgCl_2_, 0.991 g glucose, 0.5957 g HEPES at PH 7.4. Following the initial observation with Fura-2AM across the various excitation spectra, we made adjustments for any inherent background luminescence. Subsequent to 5 minutes post-observation, calcium ions at a concentration of 2 mM were introduced into the solution, which then underwent a 10-min incubation period. A fluorescence microscope system (Nikon, Japan) was used for fluorescence signal detection. To quantify alterations in the calcium ion concentration, we computed the emitted fluorescence ratio (F0/F1).

### Statistical analysis

Data were analyzed statistically through GraphPad Prism 6.0 (USA), with values depicted as means ± SD. Multiple group comparisons were conducted via one-way ANOVA, while pair-wise comparisons relied on the Student's t-test. Statistical significance was established at *p* < 0.05. Each experiment was repeated at least 5 times, and each repeat was performed as a separate, independent experiment or observation (Panos and Boeckler, 2023).

## Results

### The optimal concentrations of PAN, RTX, and MP for podocytes

**FIGURE 1:**
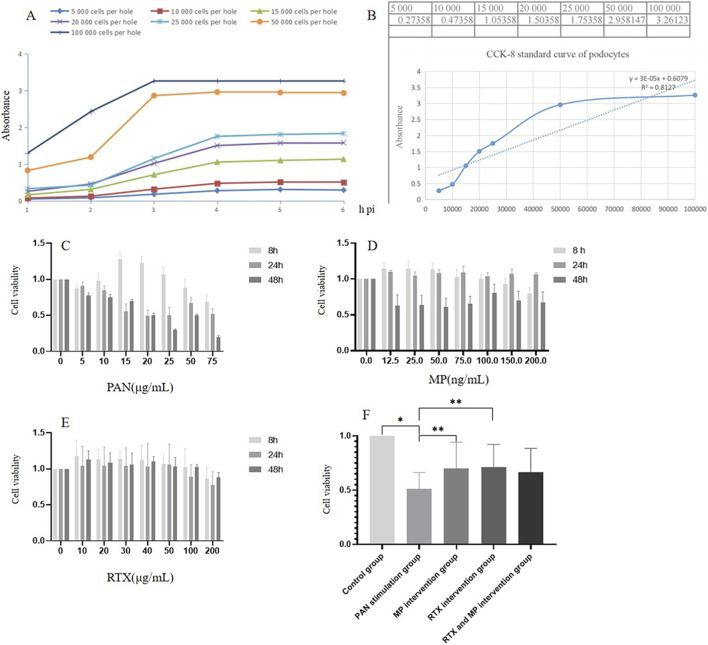
Effects of MP, RTX, or PAN on MPC5 podocyte stimulation. **(A)** Cell activity in different number of podocytes. **(B)** Cell survival rate of different number of podocytes for 4 h **(C–E)** Effects of PAN, MP, and RTX concentration on podocyte viability at different time points. **(F)** Effects of the optimal MP or RTX concentration on the opitmal PAN concentration on podocyte viability for 48 h (N = 5). (Compared with control group, *p < 0.05; compared with PAN stimulation group. **p < 0.05).

An inverted microscope was utilized to examine and capture images of the cells. The dilution concentration of each cell line was stable when CCK-8 was added for 4 h (Figure 1A). Hence, a 4-h duration was employed for the construction of the reference curve, as depicted in Figure 1B. The number of 20,000 and 50,000 cells in the 96-well plate were more accurate than those in the others. At 8 h, 24 h, and 48 h, CCK-8 assay found that PAN (50 μg/mL) had actual cell viability percentages and optical density (OD) values, and it could be used as the optimum concentration to induce podocyte injury (Figure 1C); MP (100 ng/mL) and RTX (100 μg/mL) maintained cell viability and had minimal impact on cell morphology, thus they were the best concentrations (Figures 1D,E). The CCK-8 assay revealed a notable reduction in cell viability within the group exposed to 50 μg/mL of PAN over a 48-h period, when contrasted with the control group, with the observed disparity reaching statistical significance (*p* < 0.05). MP (100 ng/mL) and RTX (100 μg/mL)-treated PAN-damaged podocytes showed that cell variability in the MP, and the RTX intervention groups were significantly higher than that in the PAN stimulation group (*p* < 0.05) (Figure 1F).

### The rate of podocyte cell death via apoptosis following injury from PAN and subsequent treatment with RTX or MP

**FIGURE 2:**
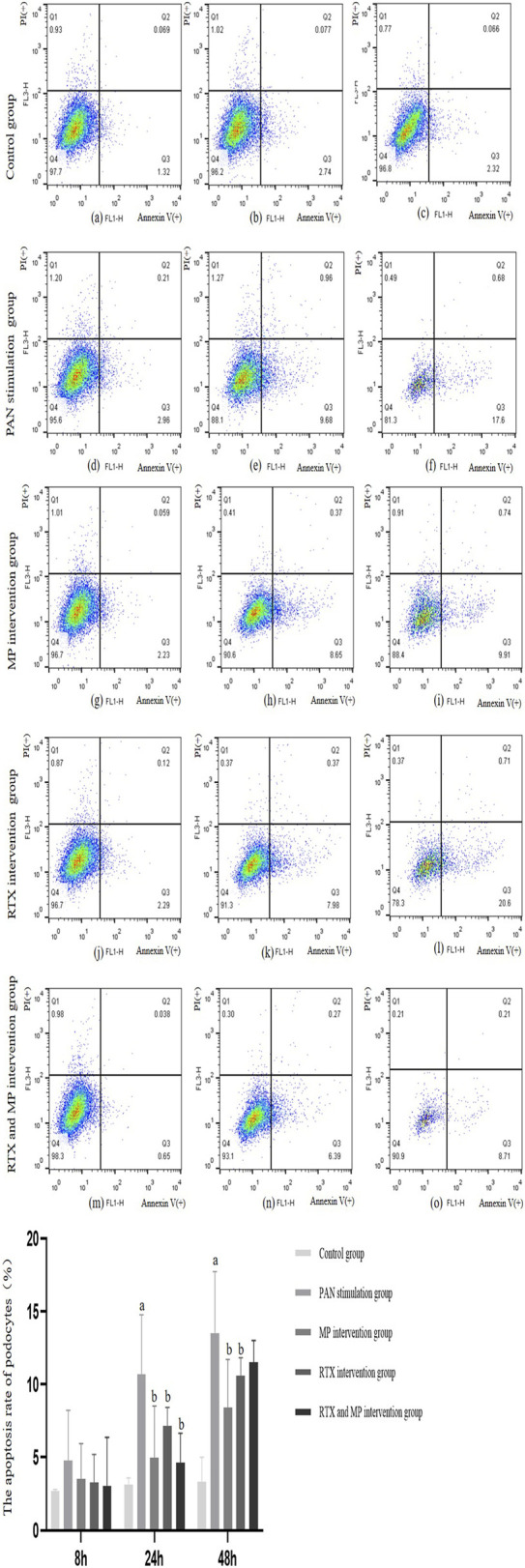
Differences in the podocyte apoptosis rate in noraml and under different drug interventions of PAN, MP, or RTX at 8 h **(a,d,g,j,m)**, 24 h **(b,e,h,k,n)**, and 48 h **(c,f,l,i,o)**. Q1 was the necrotic cell, Q2 was the late apoptic cell, Q3 was the early apoptic cell, and Q4 was the number of viable cells. The apoptosis rate of podocytes was the sum of Q2 and Q3. (N = 5). (Compared with control group, ^a^ *p*<0.05; compared with PAN stimulation group, ^b^ *p*<0.05).

Flow cytometric analysis, employing propidium iodide (PI) and Annexin V-FITC dual staining detection kits, revealed the apoptosis levels in podocytes. The findings demonstrated that, following 8 h of PAN exposure, podocyte apoptosis frequencies did not differ markedly from those in the standard control group (*p* > 0.05). Conversely, the incidence of podocyte apoptosis at 24 and 48 h post-PAN treatment were significantly elevated when measured against the control group (*p* < 0.05). At both 24 and 48-h intervals, the podocyte apoptosis frequencies in the groups treated with RTX or MP were notably reduced about 30%–50% compared to the group stimulated with PAN, with the differences reaching statistical significance (*p* < 0.05) as depicted in Figure 2.

### TRPC6 messenger RNA (mRNA) expression changes

**FIGURE 3:**
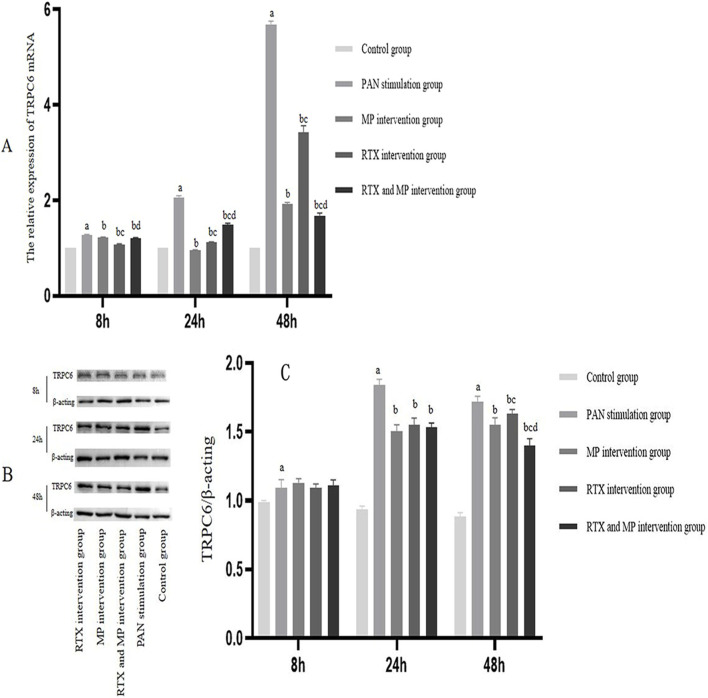
Relative expression levels of TRPC6 mRNA and different proteins in podocytes. **(A)** Expression of TRPC6 mRNA in control, PAN, MP, RTX, and RTX and MP podocytes at different time points. **(B)** Expression of TRPC6 and β-actin protein in control, PAN, MP, RTX, and RTX and MP podocytes at different time points. **(C)** The ratio of TRPC6 protein expression to the internal reference β-actin. (N = 7). (Compared with control group, ^a^ *p*<0.05; compared with PAN stimulation group, ^b^ *p*<0.05; compared with MP intervention group, ^c^ *p*<0.05; compared with RTX intervention group, ^d^ *p*<0.05).

Under normal conditions, podocytes express TRPC6 mRNA. Employing GAPDH as a reference standard, there was a notable elevation in TRPC6 mRNA levels following exposure to PAN for 8, 24, and 48 h (*p* < 0.05). In contrast, treatments with MP, RTX, and combined RTX and MP for similar time frames resulted in markedly greater reductions of 10%–60% in TRPC6 mRNA levels compared to those observed in the PAN-treated cohort (*p* < 0.05). Moreover, comparative analysis revealed that TRPC6 mRNA levels were substantially reduced in the RTX-treated group relative to the MP-treated group at the 8-h mark. Nonetheless, the levels of TRPC6 mRNA observed within the RTX treatment cohort exhibited a marked increase compared to the MP treatment cohort at 24 and 48 h (*p* < 0.05). The presence of TRPC6 mRNA within the cohort treated with RTX and MP was elevated relative to the RTX group at the 8-h and 24-h marks, yet it declined below the level observed in the RTX group at the 48-h mark (*p* < 0.05) (see Figure 3A).

### TRPC6 protein expression changes

Results from western blotting indicated the presence of distinct bands for TRPC6 and β-actin within the molecular weight ranges of 100–130 kDa and 35–55 kDa, respectively. Typically, podocytes exhibit a baseline expression level of TRPC6 protein. Relative to the baseline control, the levels of TRPC6 protein saw a notable rise following PAN treatment at 8, 24, and 48-h intervals (*p* < 0.05); however, when assessing the groups subject to MP, RTX, and the combined RTX and MP interventions against those just given PAN, TRPC6 protein levels displayed no marked changes at the 8-h mark and exhibited reductions of 5%–20% after both 24 and 48 h (*p* < 0.05). After 48 h, TRPC6 levels were markedly reduced in the RTX and MP group compared to the MP group alone, while the RTX and MP group also displayed a substantial decrease in TRPC6 when contrasted with the RTX group alone (*p* < 0.05) (Figures 3B,C).

### IL-1β and IL-18 levels in podocytes culture supernatant changes

**FIGURE 4:**
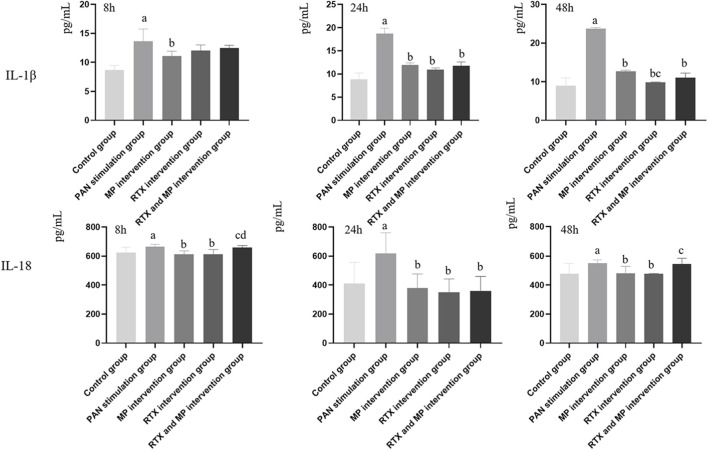
Levels of IL-1β and IL-18 in podocytes culture supernatants in control, PAN, MP, RTX, and RTX and MP podocytes at different time points. (N = 5) (Compared with control group, ^a^ *p*<0.05; compared with PAN stimulation group, ^b^ *p*<0.05; compared with MP intervention group, ^c^ *p*<0.05; compared with RTX intervention group, ^d^ *p*<0.05).

Following PAN stimulation, the concentrations of IL-1β and IL-18 were notably elevated compared to the normal control group at 8, 24, and 48 h intervals (*p* < 0.05). When compared to the PAN treated cohort, the group receiving MP intervention exhibited substantially reduced quantities of IL-1β and IL-18 at the same time points (*p* < 0.05). Additionally, the RTX intervention led to significant reductions in IL-1β and IL-18 concentrations relative to the group subjected to PAN stimulation at both 24 and 48 h measurements. Significantly reduced IL-1β concentration was observed in the group treated with RTX and MP compared to the group subjected to PAN stimulation at 24 and 48 h, as indicated by a *p*-value less than 0.05. IL-18 level in the RTX and MP intervention group was significantly lower than that in the PAN stimulation group after 24 h (*p* < 0.05) (Figure 4).

### TRPC6 distribution changes in podocytes

**FIGURE 5:**
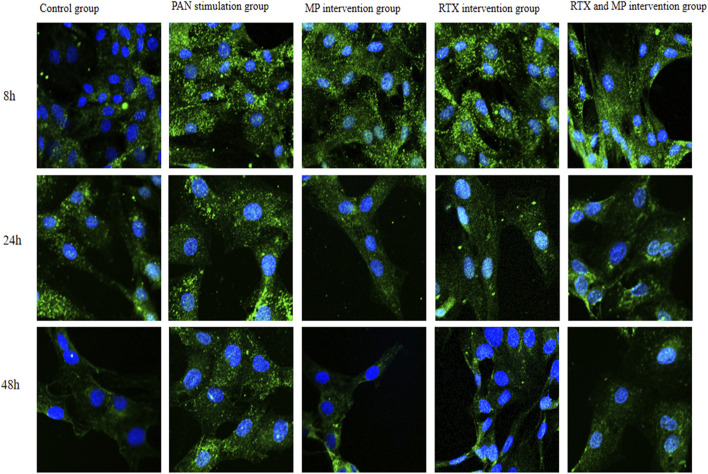
Effects of PAN, MP, or RTX on the distribution and protein expression of TRPC6 at different time points. Efficiency of TRPC6 expression in cultured podocytes was determined by laser scanning confocal microscope (63×, oil immersion lens). TRPC6 was labeled green with FITC filterat a 488 nm excitation wavelength, podocyte nuclei were stained blue with DAPI. (N = 5).

Analysis via immunofluorescence revealed a consistent and linear pattern of TRPC6 localisation within the plasma membrane of the control cells, with a minimal cytoplasmic presence; conversely, following exposure to PAN for 8 and 24 h, the TRPC6 presence became patchy at the plasma membrane with a notable rise within the cytoplasm. Post 48 h of PAN exposure, there was an upsurge of TRPC6 at specific regions of the plasma membrane, with some areas exhibiting a loss of TRPC6, which aggregated into granule-like formations and exhibited extensive cytoplasmic distribution. Following intervention with MP or RTX, the distribution of TRPC6 across the cell membrane became more homogenous at various time intervals, and there was a notable enhancement in its distribution throughout the entire cell, approaching a normal pattern as depicted in Figure 5.

### Calcium imaging

**FIGURE 6:**
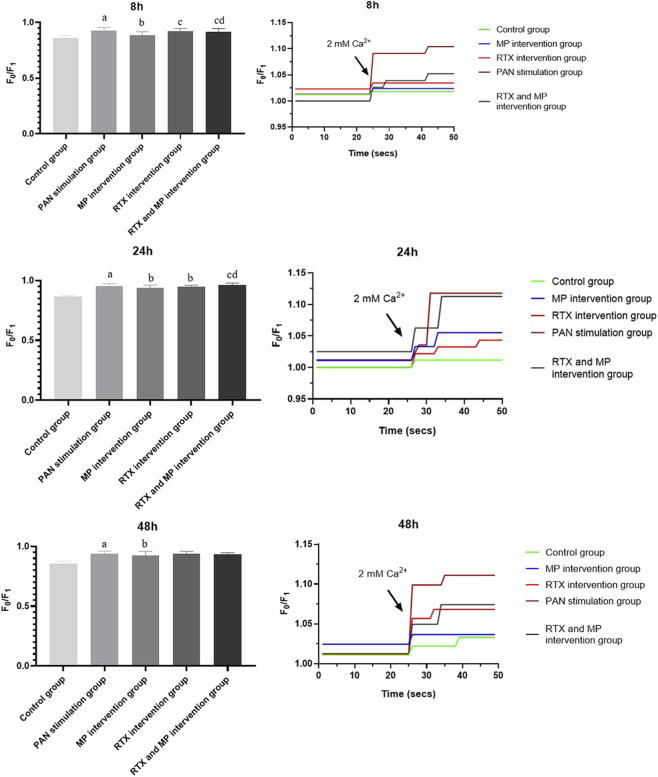
Differences in Ca^2+^ influx in control and under different drug intervetions of the PAN, MP, or RTX at different time points. (N = 5). (Compared with control group, ^a^ *p*<0.05; compared with PAN stimulation group, ^b^ *p*<0.05; compared with MP intervention group, ^c^ *p*<0.05; compared with RTX intervention group, ^d^ *p*<0.05).

To better understand whether the induction of TRPC6 is associated with changes in intracellular Ca^2+^, we examined Ca^2+^ influx in cultured podocytes after PAN injury. Following PAN exposure in cultured podocytes, heightened Ca^2+^ entry was noted at intervals of 8, 24, and 48 h when compared to controls (*p* < 0.05), indicative of its role in podocyte damage, potentially via TRPC6 channel activation as depicted in Figure 6. Additionally, at these same time points, the group treated with MP exhibited a reduction in Ca^2+^ influx relative to the group subjected to PAN (*p* < 0.05). At 8 h, the RTX intervention group had a higher Ca^2+^ influx than the MP intervention group (*p* < 0.05), and at 24 h, the RTX intervention group had a lower Ca^2+^ influx than the PAN stimulation group (*p* < 0.05). At 8 and 24 h, the combined RTX and MP intervention group had a higher Ca^2+^ influx than the MP and the RTX intervention groups (*p* < 0.05).

## Discussion

Podocytes, are specialized cells within the GFB, which are crucial for maintaining glomerular structural integrity and convective ultrafiltration (Loreth et al., 2025). Podocyte dysfunction, resulting from oxidative stress, dysregulated prosurvival signaling, or structural damage, can drive the development of proteinuria and glomerulosclerosis (Loreth et al., 2025; Haydak and Azeloglu, 2024). Functionally, podocyte injury leads to actin cytoskeleton rearrangement and the merging and disappearance of FPs, and dysregulates SD protein expression, which could induce podocyte depletion and impair ultrafiltration (Meliambro et al., 2024). The mechanism of PAN associated podocyte injuries was that it could trigger the effacement of podocyte FPs, resulting in cytoskeletal disruption and atypical expression and allocation of podocyte molecules (Ding et al., 2021; Qiu et al., 2021; Huang et al., 2025). The current research constructed a model of podocyte damage utilizing PAN, and showed that the numbers of podocytes were markedly reduced in the PAN stimulation group following 48 h of exposure to PAN, and the apoptotic rates were notably elevated after 8, 24, and 48 h of PAN treatment, indicating that PAN can lead to podocyte depletion. Moreover, following treatment with MP, and RTX over a 48-h period, there was a notable rise in podocyte counts, and when applied for both 24 and 48 h, these agents markedly reduced the apoptosis rates, suggesting that MP, and RTX might be possible therapeutic implications in podocytes depletion.

The increased expression and activity of TRPC6 leads to aberrant cytoskeletal rearrangements in podocytes, podocyte FPs effacement, and eventually podocyte death (Ding et al., 2021; Qiu et al., 2021; Hart et al., 2023). A gain-of-function mutation in TRPC6 was identified as monogenic cause of FSGS, however, a small number of mutations with a loss-of-function TRPC6 phenotype have also been associated with FSGS (Riehle et al., 2016) and a novel heterozygous loss-of-function TRPC6 mutation was not associated with FSGS (Batool et al., 2023). TRPC6 expression was also increased in non-hereditary proteinuric kidney disorders (Salemkour et al., 2023; Möller et al., 2007), indicating that it can be targeted for treatment. Nevertheless, in the current study, elevations in levels of TRPC6 mRNA and protein following exposure to PAN for durations of 8, 24, and 48 h, surpassed those observed in the control set. These findings align with previously published studies (Tu et al., 2023; Ma et al., 2024). Concurrently, the data from our experiments further revealed that post-intervention with MP or RTX, there were diminished expressions of TRPC6 at corresponding time intervals at both the mRNA and protein levels, suggesting that MP’s or RTX’s ability to lessen the damage PAN causes to podocytes.

The primary therapies for many glomerular diseases are glucocorticoids, which exert their immunosuppressive and direct podocyte protective effects via the glucocorticoid receptor (Agrawal et al., 2021). Podocyte-targeted delivery of TRPC6 short-interfering RNA using an antibody delivery system reduced podocyte TRPC6 expression in rats, and TRPC6 short-interfering RNA prevented AngII-induced apoptosis and increased markers of autophagy in cultured mouse podocytes (Feng et al., 2022). Recently, in an adriamycin-induced mouse nephropathy model, TRPC6-targeted Dex-loaded nanobubles (Dex@NBs), administered at half the dosage of free Dex, markedly alleviated proteinuria, glomerular and tubular damage, renal apoptosis, inflammation, and fibrosis (Wu et al., 2025), which were aligned with our findings, enhancing MP’s organization within the cells and reducing both mRNA expression and protein dispersion during PAN-induced podocyte damage featuring elevated TRPC6 expression.

The kidney is an important organ for the maintenance of Ca^2+^ homeostasis in the body (Semenikhina et al., 2023). Enhanced Ca^2+^ entry stimulates the development of actin-myosin contractility along with stress fibers in the cellular structure, which, when activated improperly, can induce architectural disarray of FPs and damage or even kill podocytes, cause the onset of various renal disorders (Hart et al., 2023; Tu et al., 2023). During the progression of kidney disease, Ca^2+^ signaling plays a key role in various cell activities such as necrosis, apoptosis, eryptosis, and autophay (Zhang et al., 2021; Semenikhina et al., 2023). *In vivo* and vitro, inhibiting TRPC6 expression could alleviate Ca^2+^ influx and the degradation of podocyte structural proteins, and reduce podocyte injury and proteinuria excretion ['t Hart et al., 2021; Ding et al., 2021; Feng et al., 2022]. Podocytes express large conductance Ca^2+^-activated K^+^ channel (BK channels) increasing Ca^2+^ influx via TRPC6 channels and KCa1.1 subunits interacting directly with TRPC6 channels in PAN-induced podocytes damage (Kim et al., 2024). Furthermore, our research confirmed that TRPC6 overexpression can activate Ca^2+^ influx in PAN-induced podocyte injury, and MP could decrease Ca^2+^ influx for 8, 24, and 48 h, whereas RTX decreased Ca^2+^ influx for 24 h. Accordingly, we propose that MP and RTX can reduce Ca^2+^ influx by inhibiting TRPC6 expression, stabilizing the number of podocytes, further protecting podocytes, and decreasing proteinuria excretion (Ning et al., 2021). Therefore, altering Ca^2+^ signaling pathways may serve as a viable therapeutic approach for diseases linked to podocytes.

This study provides evidence that following PAN stimulation, the concentrations of IL-1β and IL-18 were notably elevated compared to the normal control group at different time intervals. When compared to the PAN treated cohort, the group receiving MP intervention exhibited substantially reduced quantities of IL-1β and IL-18 at the same time point, while the RTX intervention led to significant reductions at both 24 and 48 h. Levels of IL-1β at 24 and 48 h and level of IL-18 at 24 h were lower in the group treated with RTX and MP compared to the group subjected to PAN stimulation. In addition, the maturation and secretion of pro-inflammatory cytokines IL-1β and Il-18 were triggered by Nod-like receptor protein 3 (NLRP3) inflammasome activation, which was induced by the increase of intracellular calcium *in vitro* and *in vivo* studies (Yang et al., 2019; Werner and Wagner, 2023). Moreover, *in vitro*, knockout of TRPC6 could decrease NLRP3 expression and intracellular Ca^2+^ concention and suppress the release of IL-1β and Il-18 in macrophages (Chen et al., 2025). TRPC6 knockout in type 2 diabetes mellitus induced hepatic inflammation and fibrosis, inhibited calcium overload, and suppressed the calcineurin/nuclear factor of activated T cells 2/NLRP3 signaling pathway in mice (Liu et al., 2025). These studies showed that NLRP3 inflammasome could be activated via the TRPC6/Ca^2+^/NLRP3 pathway, contributing to inflammation, concurring with our findings that the expression of TRPC6 and its channel could promote calcium influx in podocytes, stimulate inflammatory agents, cause podocyte injury, and release IL-1β and Il-18.

This *in vitro* study also confirmed that a common distribution existed between MP and RTX ligand on TRPC6; thus, we inferred that MP and RTX might interact with TRPC6. Moreover, in the current study, MP and RTX treatment decreased the expressions of TRPC6 mRNA and protein at 24 and 48 h, respectively, but increased Ca^2+^ influx at 24 h, suggesting that the Ca^2+^ signal network may participate in the regulation of podocyte injury, and TRPC6 might mediate extracellular Ca^2+^ influx. Following a period of 48 h, levels of TRPC6 mRNA and protein were found to be diminished in combined RTX and MP intervention as compared to those observed in the MP and the RTX intervention. Nonetheless, at intervals of 8 and 24 h, there was a noticeably increased intake of Ca^2+^, leading us to surmise that different channels or regulatory molecules could be involved in the damage to podocytes. Additionally, there are at least two other TRPC channels, such as TRPC3 and TRPC5, expressed in podocytes. The TRPC3 channels cannot be activated by application of ATP in the absence of TRPC6 (Staruschenko et al., 2023). Untill now, TRPC5 expression couldn’t compared with TRPC6 in human renal biopsies, moreover, TRPC5 plays a role redundant to that of TRPC6 in podocytes (Staruschenko et al., 2023; Polat et al., 2023). Concurrently, it is imperative to conduct more extensive research into the precise molecular processes and to corroborate these findings through further examinations employing TRPC6 inhibitors, as indicated by our research or subsequent studies utilizing pertinent animal models (Hart et al., 2023; Tu et al., 2023; Batool et al., 2023).

## Conclusion

In conclusion, our study indicates that both MP and RTX have the potential to diminish apoptotic rates and maintain podocyte counts, achieved through suppression of excessive TRPC6 expression, enhancement of TRPC6 arrangement within podocytes, reduction of calcium entry, and mitigation of PAN’s detrimental impact on these cells. These could offer foundational rationales for the therapeutic employment of MP, and RTX in renal pathologies. Collectively, the findings imply a contributory factor of TRPC6 in the harm to podocytes via the disruption of the calcium signaling cascade.

## Data Availability

The original contributions presented in the study are included in the article/supplementary material, further inquiries can be directed to the corresponding author.
